# Characterization of spatial lipidomic signatures in tick-bitten guinea pig skin as a model for host-vector-pathogen interaction profiling

**DOI:** 10.1128/msystems.00927-23

**Published:** 2023-10-24

**Authors:** Alison J. Scott, Alexis A. Smith, Ron M. A. Heeren, Utpal Pal, Robert K. Ernst

**Affiliations:** 1 Department of Microbial Pathogenesis, University of Maryland, Baltimore, Maryland, USA; 2 Maastricht MultiModal Molecular Imaging (M4i) Institute, Maastricht University, Maastricht, Limburg, the Netherlands; 3 Department of Veterinary Medicine, University of Maryland, College Park, Maryland, USA; Pacific Northwest National Laboratory, Richland, Washington, USA

**Keywords:** vector-borne diseases, *Ixodes*, guinea pig, spatial lipidomics, lipids, host-vector interaction

## Abstract

Spatially aware *de novo* discovery methods are essential tools for therapeutic target identification in complex interphylum interactions such as arthropods and mammals. Notably, the methods should ideally be species agnostic, showing unique features of all interacting species. We evaluated the possibilities for matrix-assisted desorption/ionization mass spectrometry imaging (MALDI-MSI, referred to here as MSI) as a spatial “omics” method to simultaneously profile both an arthropod vector (*Ixodes* tick) and a mammalian skin (guinea pig) in a bite model. We demonstrated the feasibility of MSI using gelatin-stabilized sample mounting that allowed for serial sectioning and mapping lipids in control and bitten skin, including the tick body and embedded mouthparts. We identified unique lipid ion patterns and observed lipid reorganization beneath the bite site consistent with histological changes. Furthermore, several ions were observed in the tick body with lower intensity in the dermis and control skin, suggesting the transmission of lipids from the tick to mammalian skin. These results establish a multi-system approach for discovering cross-species molecular interactions that can be further developed as targets to disrupt the vector-host interface.

**IMPORTANCE:**

Here, we demonstrate the adaptability of spatial “omics” methods to identify interphylum processes regulated at the vector-host interface of ticks during a mammalian blood meal. This approach enables a better understanding of complex bipartite or tripartite molecular interactions between hosts, arthropod vectors and transmitted pathogens, and contributes toward the development of spatially aware therapeutic target discovery and description.

## OBSERVATION

Mass spectrometry imaging (MSI) presents new opportunities for mechanistic discovery in host-pathogen and vector-host interactions (1–4). Notable examples include arthropod vectors biting host skin (mosquitos, ticks, and fleas), a common mode of intradermal disease transmission (5). Despite extensive research, the complex interface formed by a tick biting mammalian skin remains poorly understood (6). The initial phase of the tick bite consists of mouth parts penetrating the epithelium, followed by remodeling of the underlying skin (7). In the later bite phase, the skin is conditioned for bacterial transmission, resulting in microbial transmission (8). The vector-pathogen interaction influences infection transmission efficiency and is an intriguing target for anti-infective strategy development (9). Preparing samples from the interphylum interaction interface can present technical challenges. Under optimal sampling, the tick body, mouth parts, and host skin layers would all be present in a thin section for the analysis by MSI. Complete embedding of a tick-bitten skin biopsy makes it challenging to target the section plane meeting those criteria. This study aimed to evaluate the potential use of pathogen-vector-host interactions for multi-system interaction imaging in a model for vector-borne disease transmission.

### Guinea pig bite model

Adult female Hartley guinea pigs were housed with food and water available *ad libitum*. Uninfected nymphal *Ixodes scapularis* were placed on guinea pigs and biopsied with the bite area before the blood meal (48 hours). Skin biopsies were collected post-mortem from both dorsal bitten skin (embedded tick) and ventral control skin (unbitten) (10).

### Sample preparation

Samples were frozen on a liquid nitrogen float and mounted (unembedded) with 4% porcine gelatin media to create a support. Cryosections were cut (12 µm) on a Leica CM 1950, thaw-mounted on indium tin-oxide slides, sealed, and stored at –80°C. Slides were thawed in a vacuum desiccator for 5 min. Norharmane (NRM) matrix was prepared in 2:1 (vol:vol) chloroform:methanol at 10 mg/mL and sonicated for 1 min in a water bath sonicator. Slides were coated with NRM matrix (both positive and negative modes) using an HTX TM sprayer with the following settings: 14 passes at 0.1 mL/min, then 2 passes at 0.04 mL/min, 1,200 mm/min, 2.5 mm track, CC pattern, 10 psi, 2 L/min, 30°C nozzle, with 2 s dry time at 40 mm height (11), and vacuum desiccated for 5 min. Matrix was removed with 70% ethanol (two dips at 30 s), stained with hematoxylin and eosin (H&E) (11), and brightfield images collected on a Zeiss AxioImager M2.

### Data capture and analysis

Data were collected on a 9.4T MALDI-FT-ICR-MS solariX instrument (Bruker Daltonics; Cryogenics) calibrated to red phosphorus using a quadratic function for positive and negative modes. Images were collected at 100 µm step size over a mass range of *m/z* 345–1,700 (neg.) and 300–2,000 (pos.) with 0.5 s transients (both modes, resolution at *m/z* 400:121,000). Full-spectrum raw data were imported into SCiLS Lab (v11.00.14179) using axes limited to *m/z* 345–1,700 neg. and 600–900 pos. using default binning with a weak (200) top hat baseline removal function and normalized (root mean squared). Feature lists of 208 negative and 413 positive peaks were generated, and images were segmented using a bisecting k-means clustering with weak denoising considering individual spectra. Segments were assigned manually using histology based on the H&E image. Raw data are available on Zenodo (12).

### Embedded tick induces epidermal and dermal changes in lipid organization

**Fig 1:**
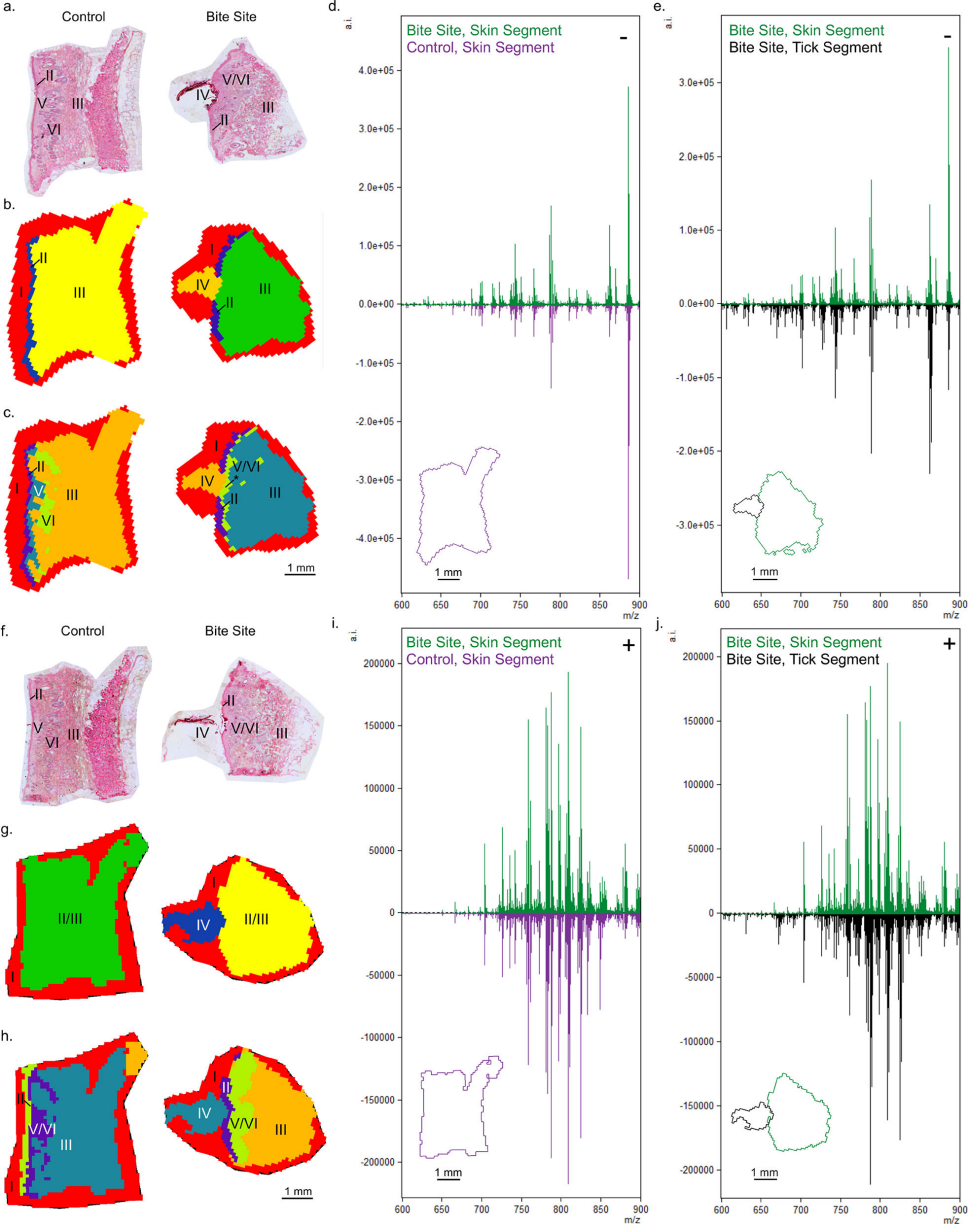
Unique lipid ion patterns mapped in tick-bitten skin distinct from control. Post-MSI histology reference (H&E) for control and bitten skin samples (a, f; 12 µm sections) oriented with epidermis facing left. Labels: I, off-tissue; II, epidermis; III, dermis and hypodermis; IV, tick; V, papillary dermis; VI, reticular dermis with hair follicles and glands. (**b**) Negative ion mode segments. (**c**) Segmentation of III colocalized to the papillary dermis and hair/secretory features of the reticular dermis. (**d and e**) Average negative spectra from control (purple), bite site skin (green), and tick body (black) segments with tissue outline shown. (**g**) Positive ion mode segments. (**h**) Segmentation of III as in panel c. (**i and j**) Average positive spectra as in panels d and e. MALDI-MSI, normalized (root mean squared[RMS]), 100 µm spatial resolution.

Negative (Fig. 1a through e) and positive (Fig. 1f through j) ion mode data sets were segmented, identifying off-tissue and on-tissue segments (Fig. 1b, c, g and h, respectively). A segment representing the tick body in the bite site skin samples was isolated from the surrounding bitten skin. In both modes, the epithelium of the control skin formed a narrow continuous line of pixels (blue, Fig. 1b through h). In contrast, the epithelium in the bite site was thickened and disrupted in negative ion mode but remained intact in the positive ion segment (Fig. 1b and c—asterisk, 1h). In negative ion mode, a segment was isolated in the papillary dermis of the control with an additional element colocalizing with the hair and secretory features in the reticular dermis (Fig. 1c). The average spectra from the control versus bitten skin segment showed changes across the phospholipid mass range in negative and positive ion modes (Fig. 1d and i). In contrast, the tick body showed distinctive ion signatures compared to the surrounding skin in both ion modes (Fig. 1e and h).

### Tick-associated features are unique from guinea pig

**Fig 2:**
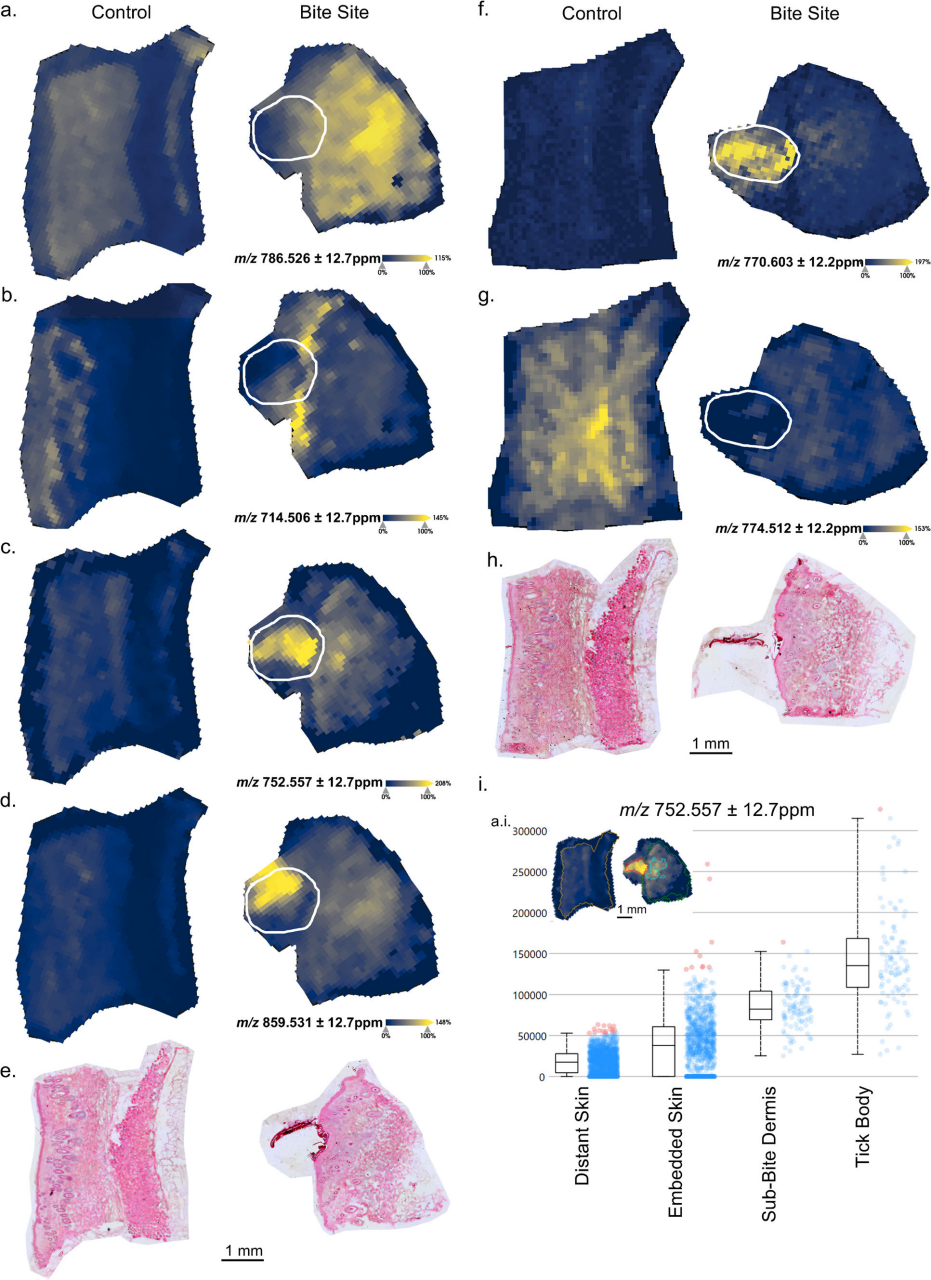
Discriminatory ion patterns from lipid images of tick-bitten skin versus control. White outline: embedded tick. (**a–d**) Negative ions, as given, and H&E (e, duplicated from Fig. 1a). (**f–g**) Positive ions, as given, and H&E (h, duplicated from Fig. 1f). (**a–d, f, and g**) Normalized (RMS) with hotspot removal and weak denoising. (**i**) Inset showing ion map from panel c with outlined regions for pixel intensity distributions on control skin, bite site skin segment, sub-bite dermis segment (cyan outline on inset), and tick body. (**a–d, f, and g**) Receiver-operator characteristic area under the curve × <0.2 or (1×) <0.2.

Tick-bitten samples showed abundant changes in spatial organization and intensity of various lipids, and the tick body resulted in a unique signature. Two examples of lipid changes were demonstrated by the negative ions *m/z* 786.526 and 714.506 ([PS 36:2-H^+^]^−^ and [PE 34:2-H^+^]^−^ all identities putative, <5 ppm) (Fig. 2a and b). The ion *m/z* 786.526 showed increased intensity in tick-bitten skin in the hypodermis, and the ion *m/z* 714.506 increased specifically in the epidermis. Interestingly, these ions were increased in tick-bitten skin but did not appear within the tick body. At the epidermal border with the tick bite, there was decreased abundance of *m/z* 714.506, suggesting interference resulting from the bite. In contrast, several ions were found in the tick body and increased in the underlying tick-bitten skin. The ion *m/z* 752.557 ([PE-O 38:4-H^+^]^−^) was found with high intensity in the tick body and within the dermal skin layer underneath the bite site (Fig. 2c). While it is not possible to determine the species origin in this experiment, we speculate that this ion is associated with the tick, possibly explaining the dermal distribution. Alternatively, the ion could be coincidentally associated with the host response. The ions *m/z* 859.531 ([PI 36:3-H^+^]^−^) and *m/z* 770.603 ([PC-O 34:0+Na^+^]^+^) showed strong intensity in the tick body with minor intensity in the bitten skin (Fig. 2d and e). Finally, a group represented by the positive ion *m/z* 774.512 ([MGDG 35:8+NH_4_^+^]^+^) showed higher intensity in the unbitten skin compared to either the tick body or the underlying bitten skin (Fig. 2f). The ion *m/z* 752.557 yielded a unique pattern in the dermis. We isolated regions control (unbitten) skin, embedded skin, tick body, and the dermal region of interest (Fig. 2g) and observed a trend between the intensity within the tick body and the dermis.

Tick bodies were stabilized and successfully sectioned with attached mammalian skin using fresh frozen samples. We collected serial sections (approximately four to five total) containing the tick and the embedded skin area. We observed structural rearrangements in the dermis; lipid signatures were associated with those histological changes. Furthermore, we identified several ion patterns enriched in the tick body compared to control or bitten skin. The most striking patterns were those ions that showed strong intensity in the tick body and appeared underneath the bite site (but were very low or absent from control skin). This is a singlet observation, and while ion identities can be approximated from accurate mass, no orthogonal methods were used, and the identities are putative. Regarding the sample preparation, most of the tissue section remained attached, but half of the exoskeleton did not remain attached to the slide—this is a sample challenge that must be addressed for future studies. Ongoing work at the host-vector interface includes finding novel mechanisms to render the bite victim less appealing for arthropod vectors, thereby preventing pathogen transmission due to the lack of a mature bite site (7). Future work will expand this method to include infected ticks to model *Borrelia burgdorferi* transmission at the bite site to understand lipids and other small molecules in vector transmission. We present a feasible starting place for future studies involving uninfected and infected ticks to simultaneously model pathogen transmission and host response.

## Data Availability

The data from this study (MSI and brightfield images) are available on Zenodo using the digital object identifiers 10.5281/zenodo.7909128 and 10.5281/zenodo.7884760, respectively.

## References

[1] Scott AJ, Flinders B, Cappell J, Liang T, Pelc RS, Tran B, Kilgour DPA, Heeren RMA, Goodlett DR, Ernst RK. 2016. Norharmane matrix enhances detection of endotoxin by MALDI-MS for simultaneous profiling of pathogen, host and vector system. Pathog Dis 74:ftw097. doi:10.1093/femspd/ftw097 27650574 PMC8427938

[2] Scott AJ, Post JM, Lerner R, Ellis SR, Lieberman J, Shirey KA, Heeren RMA, Bindila L, Ernst RK. 2017. Host-based lipid inflammation drives pathogenesis in Francisella infection. Proc Natl Acad Sci U S A 114:12596–12601. doi:10.1073/pnas.1712887114 29109289 PMC5703311

[3] Cassat JE, Moore JL, Wilson KJ, Stark Z, Prentice BM, Van de Plas R, Perry WJ, Zhang Y, Virostko J, Colvin DC, Rose KL, Judd AM, Reyzer ML, Spraggins JM, Grunenwald CM, Gore JC, Caprioli RM, Skaar EP. 2018. Integrated molecular imaging reveals tissue heterogeneity driving host-pathogen interactions. Sci Transl Med 10:eaan6361. doi:10.1126/scitranslmed.aan6361 29540616 PMC6005374

[4] Moore JL, Becker KW, Nicklay JJ, Boyd KL, Skaar EP, Caprioli RM. 2014. Imaging mass spectrometry for assessing temporal proteomics: analysis of calprotectin in Acinetobacter baumannii pulmonary infection. Proteomics 14:820–828. doi:10.1002/pmic.201300046 23754577 PMC3883928

[5] Laroche M, Raoult D, Parola P. 2018. Insects and the transmission of bacterial agents. Microbiol Spectr 6. doi:10.1128/microbiolspec.MTBP-0017-2016 PMC11633630 30306888

[6] Wikel S. 2013. Ticks and tick-borne pathogens at the cutaneous interface: host defenses, tick countermeasures, and a suitable environment for pathogen establishment. Front Microbiol 4:337. doi:10.3389/fmicb.2013.00337 24312085 PMC3833115

[7] Kitsou C, Fikrig E, Pal U. 2021. Tick host immunity: vector immunomodulation and acquired tick resistance. Trends Immunol 42:554–574. doi:10.1016/j.it.2021.05.005 34074602 PMC10089699

[8] Strobl J, Mündler V, Müller S, Gindl A, Berent S, Schötta A-M, Kleissl L, Staud C, Redl A, Unterluggauer L, Aguilar González AE, Weninger ST, Atzmüller D, Klasinc R, Stanek G, Markowicz M, Stockinger H, Stary G. 2022. Tick feeding modulates the human skin immune landscape to facilitate tick-borne pathogen transmission. J Clin Invest 132:e161188. doi:10.1172/JCI161188 36166299 PMC9621130

[9] Kitsou C, Pal U. 2021. Vaccine design, methods and protocols, volume 2. Vaccines for 213 veterinary diseases. Methods Mol biology 2411:269–286. 10.1007/978-1-0716-1888-2_16 34816411

[10] Kurokawa C, Narasimhan S, Vidyarthi A, Booth CJ, Mehta S, Meister L, Diktas H, Strank N, Lynn GE, DePonte K, Craft J, Fikrig E. 2020. Repeat tick exposure elicits distinct immune responses in Guinea pigs and mice. Ticks Tick Borne Dis 11:101529. doi:10.1016/j.ttbdis.2020.101529 32993942 PMC7530331

[11] Scott AJ, Chandler CE, Ellis SR, Heeren RMA, Ernst RK. 2019. Maintenance of deep lung architecture and automated airway segmentation for 3D mass spectrometry imaging. Sci Rep 9:20160. doi:10.1038/s41598-019-56364-4 31882724 PMC6934789

[12] Scott AJ. 2023. Zenodo. Guinea Pig Skin & Tick Bite Site - MSI Raw Data Files. Available from: 10.5281/zenodo.7909128.

